# A Systematic Review and Meta-Analysis of the Accuracy of in Vivo Reflectance Confocal Microscopy for the Diagnosis of Primary Basal Cell Carcinoma

**DOI:** 10.3390/jcm8091462

**Published:** 2019-09-13

**Authors:** Mihai Lupu, Iris Maria Popa, Vlad Mihai Voiculescu, Ana Caruntu, Constantin Caruntu

**Affiliations:** 1 Department of Dermatology, “Carol Davila” University of Medicine and Pharmacy, 050474 Bucharest, Romania; 2 Department of Plastic and Reconstructive Surgery, “Bagdasar-Arseni” Clinical Emergency Hospital, 041915 Bucharest, Romania; 3 Department of Dermatology, “Elias” University Emergency Hospital, 011461 Bucharest, Romania; 4 Department of Oral and Maxillofacial Surgery, “Carol Davila” Central Military Emergency Hospital, 010825 Bucharest, Romania; 5 “Titu Maiorescu” University, Faculty of Medicine, 031593 Bucharest, Romania; 6 Department of Dermatology, “Prof. N. Paulescu” National Institute of Diabetes, Nutrition and Metabolic Diseases, 011233 Bucharest, Romania; 7 Department of Physiology, “Carol Davila” University of Medicine and Pharmacy, 050474 Bucharest, Romania

**Keywords:** basal cell carcinoma, diagnostic test accuracy, in vivo, reflectance confocal microscopy, systematic review, meta-analysis

## Abstract

Basal cell carcinoma (BCC) is the most common cancer worldwide and its incidence is constantly rising. Early diagnosis and treatment can significantly reduce patient morbidity and healthcare costs. The value of reflectance confocal microscopy (RCM) in non-melanoma skin cancer diagnosis is still under debate. This systematic review and meta-analysis were conducted to assess the diagnostic accuracy of RCM in primary BCC. PubMed, Google Scholar, Scopus, and Web of Science databases were searched up to July 05, 2019, to collect articles concerning primary BCC diagnosis through RCM. The studies’ methodological quality was assessed by the QUADAS-2 tool. The meta-analysis was conducted using Stata 13.0, RevMan 5.0, and MetaDisc 1.4 software. We included 15 studies totaling a number of 4163 lesions. The pooled sensitivity and specificity were 0.92 (95% CI, 0.87–0.95; I^2^ = 85.27%) and 0.93 (95% CI, 0.85–0.97; I^2^ = 94.61%), the pooled positive and negative likelihood ratios were 13.51 (95% CI, 5.8–31.37; I^2^ = 91.01%) and 0.08 (95% CI, 0.05–0.14; I^2^ = 84.83%), and the pooled diagnostic odds ratio was 160.31 (95% CI, 64.73–397.02; I^2^ = 71%). Despite the heterogeneity and risk of bias, this study demonstrates that RCM, through its high sensitivity and specificity, may have a significant clinical impact on the diagnosis of primary BCC.

## 1. Introduction

A significant increase in the worldwide incidence and prevalence of skin cancer, and especially basal cell carcinoma (BCC), has been reported in recent years [1,2,3,4]. Although locally invasive, this keratinocyte carcinoma has an excellent prognosis when diagnosed and treated early.

The routine diagnosis of BCC is based on clinical evaluation and histopathological examination, however with several caveats to this practice. Clinical diagnosis relies on the experience of the dermatologist and is subject to observer bias, and histopathological examination requires an invasive procedure prone to unavoidable sampling errors [5], sometimes requiring several interventions until a final diagnosis is reached.

Multiple techniques that enable non-invasive, real-time diagnosis of skin tumors have been developed, including dermoscopy, high-frequency ultrasonography [6], optical coherence tomography, multi-modal imaging [7], and reflectance confocal microscopy (RCM) [8,9,10]. RCM enables in vivo, non-invasive imaging of the skin layers and cellular structures in a horizontal plane at quasi-histologic resolution [11]. This imaging technique has been widely used in the diagnosis [12,13,14,15,16,17,18,19,20] and therapeutic monitoring [21,22,23,24,25] of skin cancer and inflammatory [26,27,28,29,30] and infectious skin diseases [31,32,33]. Numerous studies have investigated the diagnostic accuracy of in vivo RCM for BCC.

To formulate comprehensive and up-to-date evidence-based suggestions for the rational use of RCM, we performed a systematic review and meta-analysis to evaluate its accuracy in the diagnosis of primary BCC using histopathology as the reference standard.

## 2. Materials and Methods

A systematic review and meta-analysis was conducted and the results were reported according to the Preferred Reporting Items for Systematic Reviews and Meta-Analysis (PRISMA) statement [34]. Adjustments were made as to adhere to the recommendations for reviewing diagnostic test accuracy reports [35]. Because this study did not directly involve patients, an ethics committee approval was not required.

### 2.1. Study Objective and Definition of Reference Standard 

The main objective of this systematic review and meta-analysis is to evaluate the accuracy of in vivo RCM for the diagnosis of primary BCC. A BCC diagnosis following histopathological examination of an incisional or excisional biopsy specimen was considered the reference standard.

### 2.2. Literature Search Strategy

One reviewer (ML) searched the following databases from inception till 05.07.2019: PubMed (keywords ”(basal cell carcinoma) AND confocal microscopy”), Google Scholar (keywords “basal cell carcinoma” AND “confocal microscopy” -”ex vivo” -”ex-vivo”, patents excluded), Web of Science (keywords ”TS = (confocal microscopy AND basal cell carcinoma)Timespan: All years. Indexes: SCI-EXPANDED, SSCI, A&HCI, CPCI-S, CPCI-SSH, BKCI-S, BKCI-SSH, ESCI, CCR-EXPANDED, IC.”) and Elsevier SCOPUS (keywords ”TITLE-ABS-KEY (“confocal microscopy” AND “basal cell carcinoma” )”). All references were imported and deduplicated using the reference manager EndNote (version X7, 1988–2013 Thomson-Reuters). Only articles written in English were taken into account for inclusion.

### 2.3. Eligibility Criteria

Two reviewers (ML and VMV) screened all retrieved articles by title and abstract to establish their relevance. Full-text recovery and analysis were done only for potentially eligible articles. Disagreements were settled through discussion with a third reviewer (MIP).

The established eligibility criteria were: (1) the RCM device used in the study was the VivaScope 1000 or 1500 (Lucid Technologies, Henrietta, NY, USA; Caliber I.D., Rochester, NY, USA); (2) the investigated lesions were primary BCCs, any histopathological subtype; (3) the reference standard was a diagnosis of BCC following the histopathological examination of incisional or excisional biopsy specimen; (4) sufficient data for the reconstruction of a 2×2 table or specified values for sensitivity (Sn) and specificity (Sp) were available.

We excluded from the analysis: (1) reviews, editorials, opinions, ex-vivo studies; (2) clinical cases or case series including less than 10 BCCs, in order to avoid a small studies effect; (3) studies were full-text and recovery was not possible, even after searching the available medical databases and/or contacting the corresponding authors. Studies thought to include overlapping populations were also excluded, keeping only the one with the largest number of participants. Additionally, the reference list of each study was checked to identify further relevant articles that may have been overlooked during initial screening.

### 2.4. Data Extraction and Quality Evaluation of the Studies 

One reviewer (ML) extracted the data from the included studies into a predefined form, validated by another reviewer (CC). The following parameters were extracted: the name of the first author, year of publication, country, number of participating centers, study type (prospective/retrospective), lesion type, number of investigators and their experience level (low/high), RCM device, total number of patients and lesions, patient gender and age (mean/median, years), confocal criteria employed for the diagnosis of BCC, number of true and false positives and negatives. 

All included articles were evaluated using the QUADAS-2 (Quality Assessment of Diagnostic Accuracy Studies) tool, which has a maximum score of 14 points [36]. QUADAS-2 offers a perspective over the methodological quality of a study through the assessment of four key domains: patient selection, index test (in vivo RCM), reference standard (histopathological examination), and patient flow and timing in the study. Each of these domains is evaluated for risk of bias, while the first three domains are also evaluated regarding applicability concerns.

### 2.5. Statistical Analysis and Meta-Analysis

Two-by-two tables were constructed for each RCM-based diagnosis of BCC against histopathology from incisional or excisional biopsy specimens and sensitivity, specificity and their 95% confidence intervals were visually represented using forest plots.

We used a bivariate model (hierarchical logistic regression) for the meta-analysis of sensitivity and specificity and to create the HSROC (summary receiver operating characteristic) curve. The HSROC curve illustrates sensitivity versus specificity and supplies information regarding the overall test performance across different thresholds. This model accounted for both the within- and between-study variability.

Every meta-analysis of diagnostic accuracy tests suffers from heterogeneity, attributed mainly to index test efficiency variation due to different diagnostic thresholds. Therefore, we considered the investigation of heterogeneity sources outweighs the mere demonstration of its existence [37]. Heterogeneity sources were evaluated through subgroup analyses and meta-regression using the following variables: study type (prospective/retrospective), reference standard (incisional/excisional biopsy), RCM device (VivaScope 1000/1500) and investigator experience level (low/high). Deeks asymmetry test and funnel plot were used to evaluate publication bias [38].

Data organization and statistical analyses were carried out using the software packages STATA (v13.0; StataCorp LP, Texas, USA), MetaDisc (v1.4; Unidad de Bioestadistica Clinica—Hospital Ramon y Cajal, Universidad Complutense, Madrid, Spain) and Review Manager (v5.3; Nordic Cochrane Center, Copenhagen, Denmark).

## 3. Results

### 3.1. Literature Search Results

**Figure 1:**
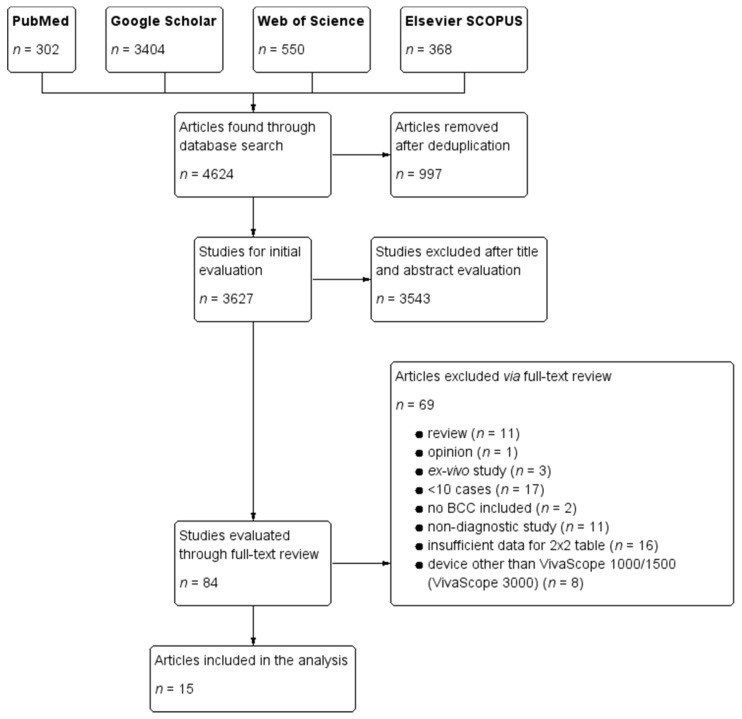
Screening process and results. Basal cell carcinoma (BCC).

The initial database search identified a total number of 4624 items. After deduplication, only 3627 remained. After title and abstract evaluation 3543 items were excluded and only 84 were selected for full-text retrieval and analysis. Sixty-nine articles were excluded based on full-text analysis (motives were recorded) (Figure 1). Fifteen studies totaling a number of 4163 lesions were included in the final analysis [5,19,39,40,41,42,43,44,45,46,47,48,49,50,51]. Study characteristics were summarized in Table 1.

The male/female ratio could not be calculated due to missing data in several studies. The manufacturer of the RCM devices VivaScope 1000 and 1500 was Lucid Inc. (Lucid Technologies, Henrietta, NY, USA), the majority of studies being carried out in Europe. A single study [39] utilized a prototype version of the VivaScope 1000 (Wellman Laboratories, Boston, MA, USA)and in two multicenter studies [41,42] different RCM devices were used, according to each participating center. Three studies did not specify the investigators’ level of experience with RCM [43,45,50]. Confocal criteria for BCC diagnosis varied considerably between studies (Table 2).

### 3.2. Quality Assessment of Study Reports

**Figure 2:**
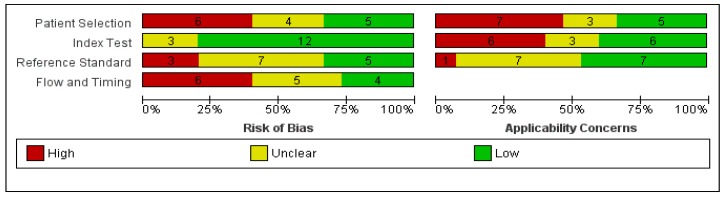
Included studies according to QUADAS-2 guidelines.

**Figure 3:**
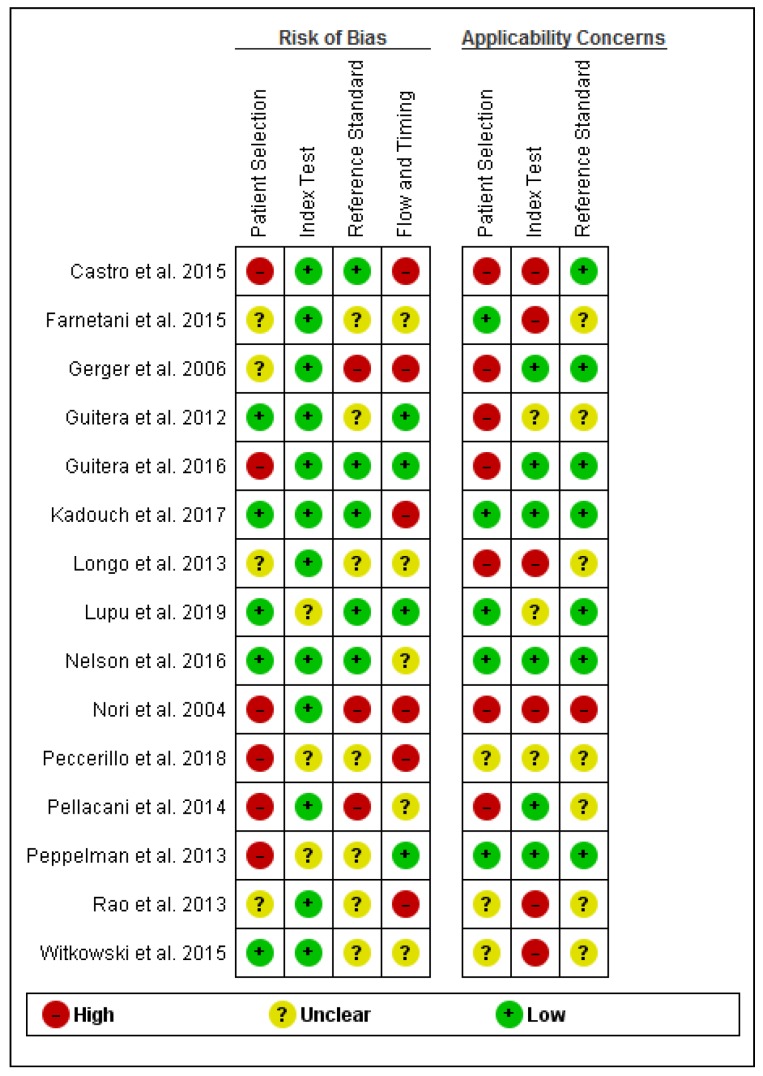
Methodological quality assessment via QUADAS-2 tool.

The results of the methodological quality assessment of the studies are illustrated in Figure 2 and Figure 3.

Eight studies had a retrospective design, while only seven were prospective. In general, the included studies exhibited high or unclear risk for bias in all domains except the index test and high or unclear applicability concerns. Ten studies (66.66%) had a high (n = 6) or unclear (n = 4) risk of bias concerning patient selection, mostly due to the exclusion of poor quality images, case-control design or unspecified patient selection protocol. Only five studies fully described the patient selection protocol. Ten studies presented high (n = 7) or uncertain (n = 3) applicability concerns owing to restrictions applied to the studied population (only including lesions highly suspicious of BCC, only including nodular lesions, etc.) and inclusion of patients with multiple lesions. In their retrospective study, Longo et al. [42] only included histopathologically confirmed nodular lesions, compensating through a relatively large sample (n = 140) and a wide variety of lesions. Peccerillo et al. [51] only included dermoscopically equivocal pigmentary lesions and excluded lesions located on the face, again compensating through a very large sample size (n = 1484). Castro et al. [46], Longo et al. [42], and Peppelman et al. [43] excluded lesions which, based on their location or the presence of hyperkeratosis, could not be evaluated by RCM and lesions in which RCM evaluation was inconclusive. Although understandable why lesions not suitable for RCM examinations due to physical limitations may not be included, these exclusions could have led to an overestimation of specificity.

Twelve out of the 15 included studies had a low risk of bias concerning the index test. More than half (n = 9) of the studies had high or uncertain applicability concerns in the index test domain due to tele-diagnosis use, blinding of the investigators to patient history or clinical data, presentation only of diagnostic consensus or lack of a diagnostic threshold.

Five studies had a low risk of bias regarding the use of the reference standard, while three were at high risk of bias owing to inadequate reference standards. Seven studies were at an unclear risk of bias. In two studies [39,40], not all lesions underwent histopathological examination. Regarding applicability concerns of the reference standard, only one study [39] had a high risk owing to the use of expert clinical diagnosis as a reference standard, while seven studies did not specify the pathologists’ experience level. Although the excision of all benign lesions included in a study is not practical, studies in which a clinical diagnosis was designated as definitive were considered as having a high risk of bias.

Regarding flow and timing according to the QUADAS-2 tool, six studies had a high risk of bias, while five and four studies had unclear and low risk of bias, respectively. Gerger et al. [40], Guitera et al. [41], Lupu et al. [19], Peccerillo et al. [51], and Longo et al. [42] included patients suspected of skin cancer (including melanoma) which could have simplified the diagnosis of basal cell carcinoma, however all studies included a fair number of both benign and malignant lesions somewhat compensating for this limitation. Nori et al. [39], Gerger et al. [40], Rao et al. [44], Peccerillo et al. [51], and Castro et al. [46] did not specify the time interval between index test (RCM) and reference standard (histopathological examination).

### 3.3. Diagnostic Accuracy of RCM and Meta-Analysis 

**Figure 4:**
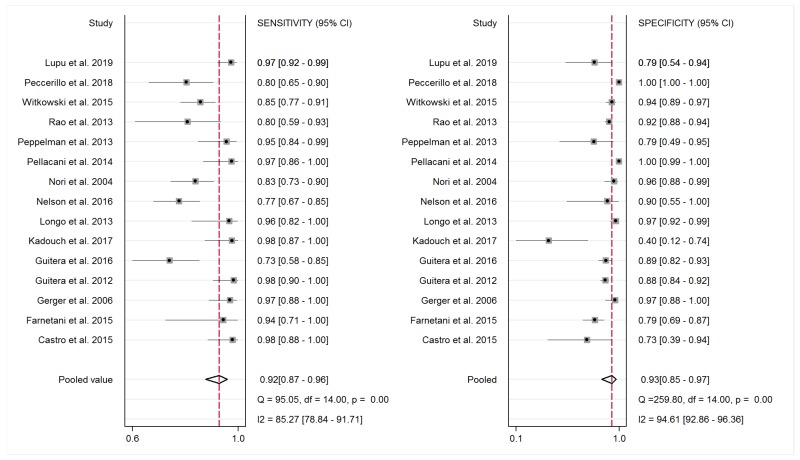
Forest plots for individual studies and pooled estimates of sensitivity and specificity with corresponding heterogeneity statistics of reflectance confocal microscopy for the diagnosis of basal cell carcinoma.

All fifteen studies were included in the meta-analysis. Sensitivity ranged from 73% to 100%, while specificity ranged from 38% to 100%. The pooled sensitivity and specificity values were 0.92 (95% CI, 0.87–0.95; I^2^ = 85.27%) and 0.93 (95% CI, 0.85–0.97; I^2^ = 94.61%). The distributions of RCM sensitivity and specificity and their summary values for the diagnosis of BCC in the included studies is represented in Figure 4.

The positive likelihood ratio ranged from 1.62 (95% CI, 0.96–2.72) to 2315.51 (95% CI, 144.33–37148.9) and the negative likelihood ratio ranged from 0.011 (95% CI, 0.001–0.17) to 0.3 (95% CI, 0.19–0.49). The pooled positive and negative likelihood ratios were 13.51 (95% CI, 5.8–31.37; I^2^ = 91.01%) and 0.08 (95% CI, 0.05–0.14; I^2^ = 84.83%). The diagnostic odds ratio (DOR) ranged from 21.37 (95% CI, 9.39–48.61) to 12725 (95% CI, 508.97–318141.1). The pooled DOR was 160.31 (95% CI, 64.73–397.02; I^2^ = 71%). 

**Figure 5:**
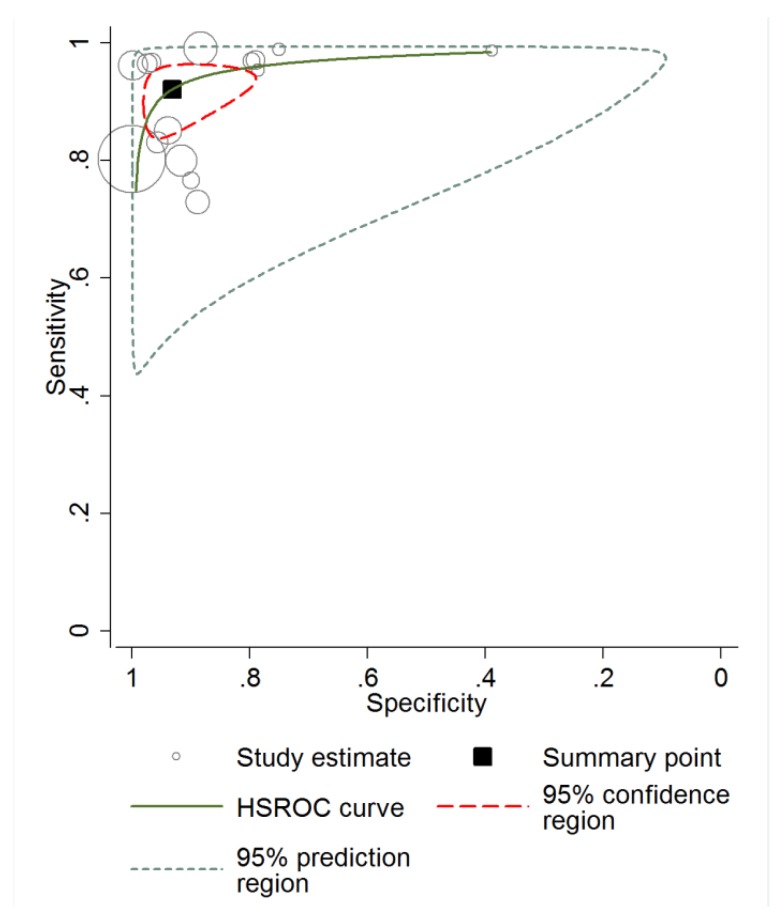
Curve summarizing reflectance confocal microscopy (RCM) sensitivity and specificity forBCC diagnosis.

The shape of the HSROC curve in Figure 5 and the area under the curve (AUC) of 0.97 suggested the lack of a threshold effect. The shape of the prediction region is meant to give a graphic representation of the extent of between-study heterogeneity, is dependent on the assumption of a bivariate normal distribution for the random effects, and should therefore not be over-interpreted [52].

### 3.4. Heterogeneity Analysis

Concerning heterogeneity analysis, a Spearman correlation coefficient of 0.468 (*p* = 0.079) suggested the lack of a threshold effect.

Next, we investigated potential sources of heterogeneity, other than the threshold effect. We performed a meta-regression analysis employing the following covariates as predictors: (1) study design (prospective/retrospective), (2) RCM device (VivaScope 1000/1500), (3) reference standard (histopathology from incisional/excisional biopsy specimen), (4) investigator experience level (low/high), and (5) number of participating centers (single center/multicenter).

The results showed that a prospective study design was associated with a 9.35 times higher RCM diagnostic performance compared with the retrospective design (RDOR = 9.35; 95% CI, 1.17;74.56; *p* = 0.037), while using the histopathology examination of the excisional biopsy specimen as a reference standard resulted in a 3.27 times (RDOR = 3.27; 95% CI, 0.93;11.47; *p* = 0.06) higher index test performance. The type of RCM device, investigator experience, and number of participating centers were not significant predictors in our meta-regression model (*p* = 0.46, 0.91 and 0.5, respectively). The results of the meta-regression are summarized in Table 3.

**Figure 6:**
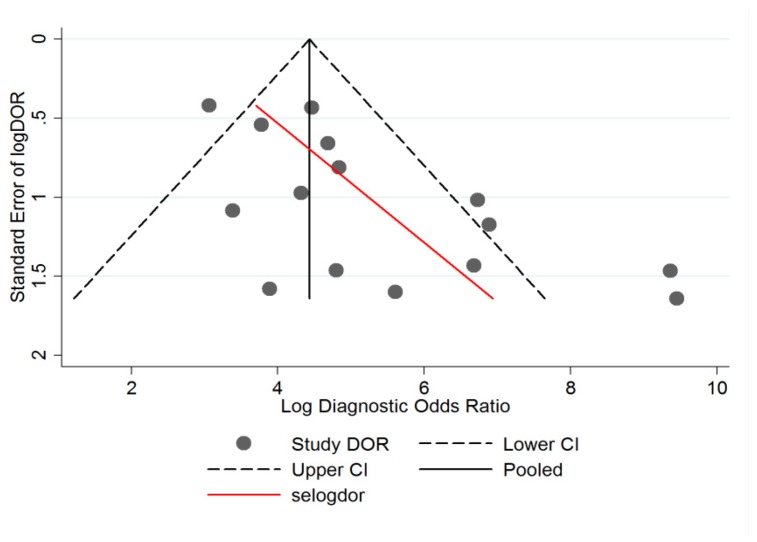
Plot with pseudo 95% confidence limits in the included studies.

Subgroup analysis revealed that RCM pooled sensitivity and specificity values in the retrospective study designs (n = 8) were 0.87 (95% CI, 0.796–0.926) and 0.95 (95% CI, 0.855–0.983) compared to 0.95 (95% CI, 0.895–0.982) and 0.90 (95% CI, 0.689–0.974) in the prospective study designs (n = 7). The pooled positive and negative likelihood ratios in retrospective studies were 17.55 (95% CI, 5.91–52.06) and 0.131 (95% CI, 0.08–0.215). The same ratios were 9.67 (95% CI, 2.73–34.27) and 0.048 (95% CI, 0.02–0.115) in prospective studies. The graphical representation of the diagnostic odds ratios (DOR) along with standard errors and confidence intervals for each study are illustrated in Figure 6.

**Figure 7:**
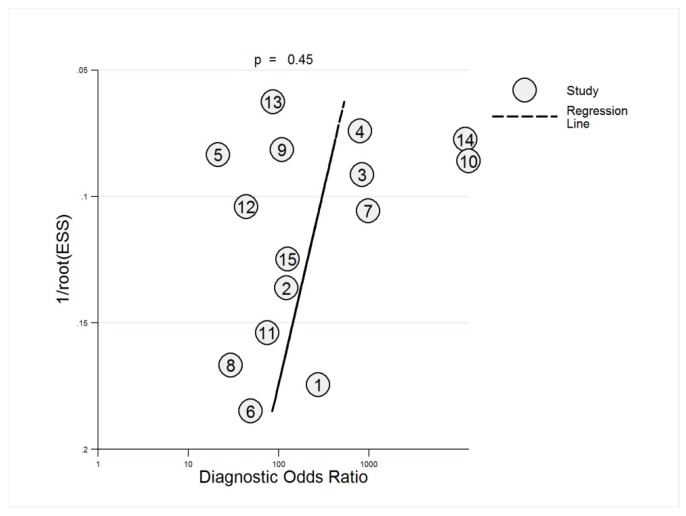
Plot of Deeks asymmetry test for publication bias.

Finally, we sought to identify potential publication bias. The funnel plot of Deeks asymmetry test [38] was relatively symmetrical (Figure 7), suggesting the lack of publication bias (*p* = 0.45).

Although we chose to report the results of the meta-analysis, they should be interpreted exercising caution and keeping in mind its limitations due to variation and potential biases.

## 4. Discussion

RCM is a novel, non-invasive diagnostic technique that enables real-time imaging of the skin down to the upper layers of the dermis at resolutions similar to histology. The confocal criteria for RCM diagnosis of various skin tumors are relatively easy to learn and the results are reproducible [53].

This systematic review and meta-analysis compares the diagnostic accuracy of RCM to histopathological examination from an incisional or excisional biopsy specimen using the results of 15 studies which included a total number of 4163 lesions. Our literature search strategy used broad keywords in multiple databases to identify as many studies as possible.

The results of the meta-analysis show a sensitivity of 92% and a specificity of 93% for the in vivoRCM diagnosis of BCC. However, these high values of both sensitivity and specificity must be interpreted with caution. The significant amount of heterogeneity renders the direct comparison of RCM diagnostic accuracy between studies impossible. RCM sensitivity for the diagnosis of BCC ranged between 73% and 100%, and its specificity ranged between 38% and 100%. Although statistically non-significant (possibly due to insufficient statistical power), these wide variations could still be attributed to the different confocal criteria and slightly different reference standards (incisional versus excisional biopsy specimen), but also investigator experience, and possibly other unknown heterogeneity sources. Investigator experience could influence diagnostic accuracy even when using the same diagnostic criteria. Rao et al. demonstrated a higher sensitivity (97.4% vs. 93.1%) and specificity (80.5% vs. 64.1%) for an investigator with over nine years of experience with RCM compared to one with only one year experience [44].

We observed that the RCM performance in prospective studies was significantly superior to that of retrospective studies (prospective vs. retrospective, RDOR = 9.35, *p* = 0.037). The pooled specificities of prospective and retrospective studies were consistent (90% vs. 95%), but the sensitivity for prospective studies was higher than that for retrospective ones (95.6% vs. 87.52%). Although the results of prospective studies were more reliable, a variety of uncontrollable factors, such as RCM devices and software and investigator experience may still influence the final diagnostic accuracy.

Subgroup analysis revealed that RCM pooled sensitivity and specificity values in the retrospective study designs (n = 8) were 0.87 (95% CI, 0.796–0.926) and 0.95 (95% CI, 0.855–0.983) compared to 0.95 (95% CI, 0.895–0.982) and 0.90 (95% CI, 0.689–0.974) in the prospective study designs (n = 7).

## 5. Clinical Relevance

**Figure 8:**
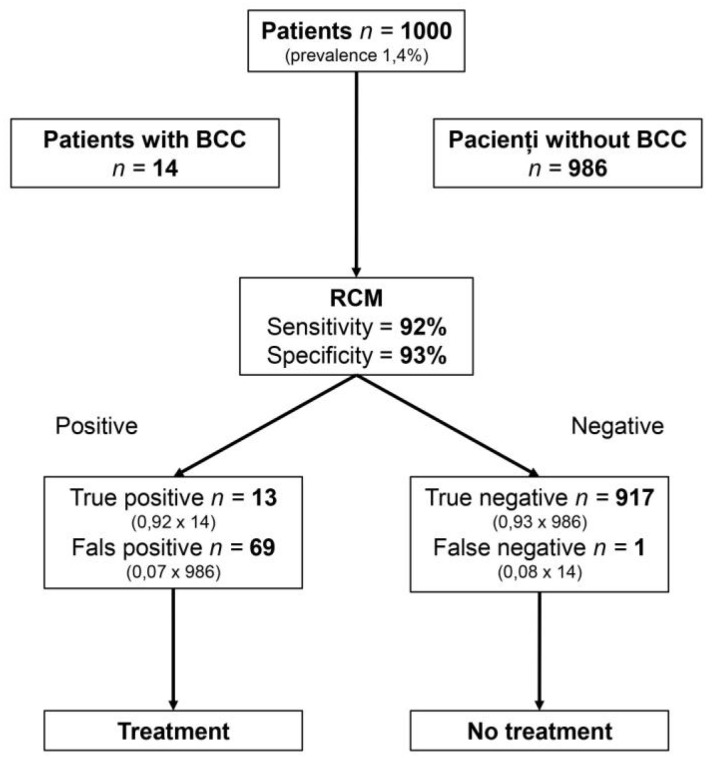
The consequences of using reflectance confocal microscopy for BCC diagnosis in a cohort of 1000 subjects. The use of RCM would lead to 82 patients being treated, of which 69 would not need to be treated; 918 patients would not be treated, of which only one would have necessitated treatment. *BCC, basal cell carcinoma; RCM, reflectance confocal microscopy.*

The results of this study may have significant implications for patients suffering from BCC. Based on recent epidemiological data, the expected prevalence of a primary BCC in Europe is 1.4% [54,55]. Using this available data together with our results, the absolute number of true and false positives and negatives can be estimated in a hypothetical cohort of 1000 subjects. This means that 14 subjects in this cohort would have a primary BCC. By using RCM as a diagnostic tool with a sensitivity of 92% and a specificity of 93%, just one of these 14 BCCs would go unnoticed, while 69 patients would be unnecessarily treated (Figure 8).

In vivo RCM could therefore become a very useful technique in the diagnosis of BCC. However, in order for it to be regarded as a potential replacement for histopathological examination, this non-invasive technique should have the ability to discriminate between the different histopathological BCC subtypes [56]. This aspect is of critical importance due to the different therapeutic approaches to BCC based on its histopathological subtype [57]. Several studies, some of which are included in this analysis [5,19,43] have sought to determine specific RCM criteria for the discrimination of BCC histotype. Unfortunately, we were unable to estimate sensitivity and specificity of BCC subtyping through in vivo RCM from the data available in the included studies.

## 6. Strengths and Limitations

We consider the adherence to the PRISMA guidelines [34], the rigorous examination of the existing literature, and the use of the QUADAS-2 tool [36] for methodological quality assessmentto be strengths of our analysis.

Our results should be interpreted bearing in mind some limitations: the relatively small number of studies (n = 15) included in the analysis; the double reference standard (histopathological examination from incisional and excisional biopsy specimen; ideally, only the excisional biopsy specimen should be used); the incomplete reporting of the patient selection process in some studies; the use of different confocal criteria for the diagnosis of BCC; the variation in RCM device and investigator experience between studies. Regarding the confocal criteria for BCC diagnosis, an international consensus for use in future studies is desirable. To facilitate homogeneity, futurestudies could consider reporting investigator experience in years, number of examined lesions and/or attended courses.

## 7. Future Directions

We expect more studies investigating the diagnostic accuracy of in vivoreflectance confocal microscopy for BCC will be carried out. To promote comparability of their results, future studies should adhere to STARD guidelines [58] and use the histopathological examination of the excisional biopsy specimen as a reference standard.

Moreover, as this non-invasive technique becomes more widely disseminated, studies could benefit from the use of RCM devices with similar technical properties and standardization of imaging protocols. To assure results comparability, these studies should report the investigators’ level of experience with RCM. More studies that investigate RCM accuracy for BCC histopathological subtype are needed. Additionally, comparative studies analyzing the cost/efficiency ratio between RCM and the current standard (histopathological examination of the incisional biopsy specimen) are warranted.

## 8. Conclusions

Reflectance confocal microscopy is a promising technique in the diagnosis of primary basal cell carcinoma. A definitive conclusion could only be drawn when a higher number of studies, possibly with homogeneous methodological approach, will be available.

## Figures and Tables

**Table 1:** Characteristics of included studies.

Author, Year, [Reference]	Country	No. of Centers	Study Design	Types of Lesion	No. of Investigators	Experience Level	Reference Standard	RCM device	No.of Patients (M/F)	Age (Mean/ Median)	No. of Lesions
Castro et al. 2015 [46]	Brazil&USA	2	prospective	BCC	2	low	histopathology (incisional)	VivaScope 1500	32 (20/12)	65	54
Gerger et al. 2006 [40]	Austria	1	prospective	melanoma, BCC, nevi, SebK	4	low	clinic & histopathology (excisional)	VivaScope 1500	119 (62/57)	n/a	120
Guitera et al. 2012 [41]	Australia&Italy	2	prospective	melanoma, BCC, SCC, nevi	2	high	histopathology (excisional)	VivaScope 1000&1500	663 (354/309)	53	710
Longo et al. 2013 [42]	Italy	2	retrospective	melanoma, BCC, SCC, nevi, SebK, DF	1	high	histopathology (n/a)	VivaScope 1000&1500	140 (64/76)	50	140
Nori et al. 2004 [39]	USA&Spain	4	retrospective	BCC, various others	1	low	clinical & histopathology (incisional)	VivaScope 1000 & Wellman Laboratories prototype	145 (n/a)	n/a	152
Peppelman et al. 2013 [43]	Netherlands	1	prospective	BCC	n/a	n/a	histopathology (incisional)	VivaScope 1500	27 (16/11)	66	57
Rao et al. 2013 [44]	USA	1	retrospective	melanoma, BCC, various benign	2	low	histopathology (incisional)	VivaScope 1500	n/a	n/a	334
Pellacani et al. 2014 [45]	Italy	1	prospective	melanoma, BCC, various benign	1	n/a	histopathology (excisional)	VivaScope 1500	408	41	292
Farnetani et al. 2015 [47]	Italy	1	retrospective	melanoma, BCC, AKs, various benign	9	high & low	histopathology (n/a)	VivaScope 1500	n/a	n/a	100
Guitera et al. 2016 [48]	Australia&Italy	3	retrospective	melanoma, BCC, AKs, various benign	1	high	histopathology (excisional)	VivaScope 1500	n/a	54.8	191
Kadouch et al. 2017 [5]	Netherlands	2	prospective	BCC	2	low	histopathology (excisional)	VivaScope 1500	46	64	46
Nelson et al. 2016 [49]	USA	1	prospective	BCC	8	low	histopathology (biopsy)	VivaScope 1500	87 (65/22)	73	100
Witkowski et al. 2015 [50]	Italy	1	retrospective	BCC, melanoma, SCC, various benign	1	n/a	histopathology (n/a)	VivaScope 1500	n/a	n/a	260
Peccerillo et al. 2018 [51]	Italy	1	retrospective	BCC, melanoma, SCC, SebK, DF	2	high	histopathology (n/a)	VivaScope 1500	n/a	n/a	1484
Lupu et al. 2019 [19]	Romania	2	retrospective	BCC, SCC, AKs, Bowen’s disease, various benign	2	high	histopathology (excisional)	VivaScope 1500	87 (36/51)	68.1	123

BCC, basal cell carcinoma; SCC, squamous cell carcinoma; SebK, seborrheic keratoses; AKs, actinic keratoses; DF, dermatofibroma; n/a, not available.

**Table 2:** Criteria for the diagnosis of basal cell carcinoma in the included studies.

Author, Year, [Reference]	Reflectance Confocal Microscopic Criteria
Castro et al. 2015 [46]	hyporefractile silhouettes, tumor islands, epidermal streaming, peripheral palisading, peri-tumoral clefting, peri-tumoral collagen bundles, increased vascularization, dendritic structures
Gerger et al. 2006 [40]	increased vascularization, epidermal streaming, peri-tumoral collagen bundles
Guitera et al. 2012 [41]	epidermal streaming, dilated horizontal blood vessels, basaloid cord or nodule, epidermal shadow, glomerular vessels, non-visible dermal papillae, epidermal disarray, dendritic structures, peri-tumoral clefting, cells with visible nuclei inside tumor islands
Longo et al. 2013 [42]	epidermal disarray, ulceration or erosion, cauliflower architecture, hyporefractile silhouettes, bright filaments inside tumor islands, increased vascularization, inflammatory infiltrate
Nori et al. 2004 [39]	elongated monomorphic nuclei, inflammatory infiltrate, increased vascularization, epidermal pleomorphism
Peppelman et al. 2013 [43]	tumor islands, peri-tumoral clefting, peripheral palisading, elongated and polarized nuclei, keratinocyte atypia and spongiosis, solar elastosis, increased vascularization, inflammatory infiltrate, leukocyte rolling
Rao et al. 2013 [44]	diagnostic criteria not specified
Pellacani et al. 2014 [45]	diagnostic criteria not specified
Farnetani et al. 2015 [47]	basaloid cords, ulceration, disarray at the dermal-epidermal junction
Guitera et al. 2016 [48]	epidermal streaming, basaloid cord or nodule, peri-tumoral fibrilar polarized pattern, peri-tumoral clefting, epidermal shadow, dark nodules, dilated horizontal blood vessels, glomerular vessels
Kadouch et al. 2017 [5]	diagnostic criteria not specified
Nelson et al. 2016 [49]	tumor islands, peri-tumoral clefting, hyporefractile silhouettes, canalicular vessels, dendritic cells
Witkowski et al. 2015	diagnostic criteria not specified
Peccerillo et al. 2018 [51]	mild keratinocyte atypia, streaming epidermis, cords connected to the epidermis, dark silhouettes, peri-tumoral clefts, ulceration/erosion, tumor island size and location (epidermal or dermal), branch-like structures in tumor island, peripheral palisading, vascular morphology (linear or coiled vessels) and diameter, collagen surrounding tumor islands, solar elastosis and inflammatory infiltrates
Lupu et al. 2019 [19]	keratinocyte atypia, epidermal streaming, ulceration, cords connected to the epidermis, small tumor islands (diameter <300 m), large tumor islands (diameter >300 m), hyporefractile silhouettes, peripheral palisading, clefting, increased vascularization, “onion-like” structures, peri-tumoral collagen bundles, inflammation represented by bright dots and plump bright cells, and dendritic cells inside tumor islands

**Table 3:** Results of the meta-regression for heterogeneity sources.

Covariate	Coefficient	Standard Error	*p*	RDOR	(95% CI)
Study design	2.236	0.9	0.037	9.35	(1.17; 74.56)
RCM device	−0.838	1.09	0.46	0.43	(0,03; 5.38)
Reference standard	1.184	0.54	0.06	3.27	(0.93; 11.47)
Investigator experience	0.067	0.59	0.91	1.07	(0.27; 4.2)
Number of centers	0.561	0.79	0.5	1.75	(0.28; 10.98)

RDOR, Relative Diagnostic Odds Ratio; RCM, reflectance confocal microscopy.
